# Description of a new species of *Aphanogmus* Thomson (Hymenoptera, Ceraphronidae) that parasitizes acarivorous gall midges of *Feltiella* (Diptera, Cecidomyiidae) in Japan

**DOI:** 10.3897/zookeys.596.8472

**Published:** 2016-06-08

**Authors:** Kazunori Matsuo, Tomoko Ganaha-Kikumura, Suguru Ohno, Junichi Yukawa

**Affiliations:** 1 Biosystematics Laboratory, Faculty of Social and Cultural Studies, Kyushu University, Fukuoka 819–0395, Japan; 2 Okinawa Prefectural Agricultural Research Center, Okinawa 901–0336, Japan; 3 Entomological Laboratory, Faculty of Agriculture, Kyushu University, Fukuoka 812–8581, Japan

**Keywords:** Aphanogmus flavigastris, Feltiella acarisuga, Feltiella acarivora, taxonomy

## Abstract

In 2008–2009, we reared small ceraphronids (about 0.5 mm in body length) from cocoons that had been made possibly by two acarivorous species, *Feltiella acarisuga* (Vallot) and *Feltiella acarivora* (Zehntner) (Diptera: Cecidomyiidae) in Okinawa, Japan. Detailed morphological observation revealed that the ceraphronid was a new species of *Aphanogmus* Thomson (Hymenoptera: Ceraphronidae). We describe it as *Aphanogmus flavigastris* Matsuo, **sp. n.** Identification of the *Aphanogmus* species is essential to evaluate its possibly negative effects on the predatory activity of *Feltiella* species that have been used as control agents against tetranychid mites.

## Introduction

In 2008–2009, small (about 0.5 mm in body length) species of ceraphronids (Hymenoptera) were reared from cocoons that had been made possibly by two acarivorous species, *Feltiella acarisuga* (Vallot) and *Feltiella acarivora* (Zehntner) (Diptera: Cecidomyiidae) in Okinawa, Japan (Abe et al. 2011, Ganaha-Kikumura et al. 2012). Preliminary identification revealed that the ceraphronid was a member of *Aphanogmus* Thomson (Hymenoptera: Ceraphronidae), which contains at least 100 species worldwide (Johnson and Musetti 2004, Evans et al. 2005, Buhl et al. 2010). About 20% of them have been known as parasitoids of various insects including Cecidomyiidae (Diptera), Bethylidae, Ichneumonidae (Hymenoptera) and Cybocephalidae (Coleoptera) (Oatman 1985, Gilkeson et al. 1993, Polaszek and Dessart 1996, Evans et al. 2005). Host information for the remaining 80% has not been provided. At present, two species, *Aphanogmus floridanus* Ashmead and *Aphanogmus fulmeki* Szelényi (=*Aphanogmus parvulus* Roberti) have been known to parasitize *Feltiella* species in the Holoarctic region. The former is an endoparasitoid of *Feltiella acarivora* (Oatman 1985, Johnson and Musetti 2004) and the latter attacks *Feltiella acarisuga*, *Feltiella acarivora*, *Aphidoletes aphidimyza* (Rondani), and *Mycodiplosis* sp. (Diptera: Cecidomyiidae) (Dessart 1992).

A few taxonomic studies have focused on Japanese species of *Aphanogmus*. Ashmead (1904) first recorded *Aphanogmus* from Japan, describing *Aphanogmus hakonensis* Ashmead based on individuals collected from Hakone, Kanagawa. Polaszak and Dessart (1996) detected several cryptic species of *Aphanogmus hakonensis* and proposed the species complex of *Aphanogmus hakonensis*. Ishii (1937) reported an unidentified species of *Aphanogmus* as a parasitoid of *Cybocephalus* species (Coleoptera: Cybocephalidae) that feed on *Unaspis yanonensis* (Kuwana) (Hemiptera: Diaspididae) on citrus in Japan. Evans et al. (2005) considered that *Aphanogmus* sp. reported in Ishii (1937) was identical to *Aphanogmus inamicus* Evans and Dessart. In total, two nominal species, *Aphanogmus hakonensis* and *Aphanogmus inamicus* have been known in Japan.

Larvae of all known *Feltiella* species feed on tetranychid mites (Acari: Tetranychidae) (Gagné 1995, Gagné and Jaschhof 2014). In particular, *Feltiella acarisuga* is regarded as an important natural enemy against tetranychid mites that frequently develop pesticide resistance and cause serious damage to various agricultural products (Barnes 1933, Wardlow and Tobin 1990). Therefore, the purpose of this study is to identify the *Aphanogmus* found in Okinawa, as this is essential to evaluate its effect on mortality of *Feltiella* species.

## Material and methods

We collected more than one larva or cocoon of *Feltiella* from each collecting site in Okinawa, Japan in 2008–2009. They were kept in petri-dishes to rear *Aphanogmus* and *Feltiella* species. Adults that emerged were preserved in 75% ethanol for morphological observation. If possible, host species of parasitoid wasp should be identified by examining remnants of host insect but the male genitalia of host cecidomyiid, which is important for species identification, would not be included in the remnants. Otherwise, host species should be identified before the attack of parasitoid wasps. However, this is not always applicable under natural conditions. Therefore, we regarded host cecidomyiid to be identical to either *Feltiella acarisuga* or *Feltiella acarivora* when *Aphanogmus flavigastris* emerged from cocoons that coexisted on the same plant with either *Feltiella acarisuga* or *Feltiella acarivora*, respectively because we have seldom seen *Feltiella acarisuga* and *Feltiella acarivora* on the same plant.

For microscopic study, the ethanol-stored specimens were dried by the method described in Matsuo and Yukawa (2009). Fore wings were mounted on slides in Canada balsam using ethanol and xylene. Several specimens were gold-coated for microphotography with a JEOL JSM-5600LV scanning electronic microscope. High resolution image was taken with the methods described in Matsuo et al. (2012). Adult morphological terminology follows Mikó and Deans (2009), except for wing venation, which follows Dessart (1963). The holotype and paratypes are deposited in the collection of the Biosystematics Laboratory, Faculty of Social and Cultural Studies, Kyushu University, Japan.

## Results and discussion

### Aphanogmus flavigastris

Taxon classificationAnimaliaHymenopteraCeraphronidae

Matsuo sp. n.

http://zoobank.org/4725144C-E843-4706-8DE2-D58805F78F41

Ceraphronidae sp.: Abe et al. 2011: 277.
Ceraphronidae sp.: Ganaha-Kikumura et al. 2012: 323.

#### Etymology.

The specific name, *flavigastris*, is Latin meaning yellowish gaster, derived from the color of the female metasoma.

#### Type material.

See Table 1.

**Table 1.** A list of type specimens of *Aphanogmus flavigastris*. All specimens are kept in the collection of the Biosystematics Laboratory, Faculty of Social and Cultural Studies, Kyushu University, Japan.

Possible host	Associated plant*	Collecting site (collector**)	Host collecting	No. specimens	Notes
*Feltiella acarisuga*	*Pueraria montana*	Senbaru, Nishihara, Okinawa, Japan (SO)	22 vii 2008	1 female	Holotype
*Feltiella acarisuga*	*Pueraria montana*	Senbaru, Nishihara, Okinawa, Japan (SO)	22 vii 2008	1 male	Paratype
*Feltiella acarisuga*	*Mallotus japonicus*	Uka, Kunigami, Okinawa, Japan (SO, TGK)	1 viii 2008	1 female	Paratype
*Feltiella acarisuga*	*Mallotus japonicus*	Uehara, Ogimi, Okinawa, Japan (SO, TGK)	6 viii 2008	1 female	Paratype
*Feltiella acarisuga*	*Broussonetia papyrifera*	Gesashi, Higashi, Okinawa, Japan (SO)	21 ii 2009	1 female	Paratype
*Feltiella acarivora*	*Bauhinia variegata*	Senbaru, Nishihara, Okinawa, Japan (SO)	18 vii 2008	2 females	Paratypes
*Feltiella acarivora*	*Melanolepis multiglandulosa*	Hentona, Kunigami, Okinawa, Japan (SO)	31 vii 2008	2 males	Paratypes
*Feltiella acarivora*	*Mucuna macrocarpa*	Oku, Kunigami, Okinawa, Japan (SO, TGK)	1 viii 2008	2 females	Paratypes
*Feltiella acarivora*	*Pueraria montana*	Iramina, Yomitan, Okinawa, Japan (SO)	2 x 2008	1 female	Paratype
*Feltiella acarivora*	*Morus australis*	Kijoka, Ogimi, Okinawa, Japan (SO)	16 x 2008	1 female	Paratype

* The plant, from which *Feltiella* species were collected.

** Name of collectors. SO : Suguru Ohno , TGK : Tomoko Ganaha-Kikumura .

#### Description.

**Figure 1.**
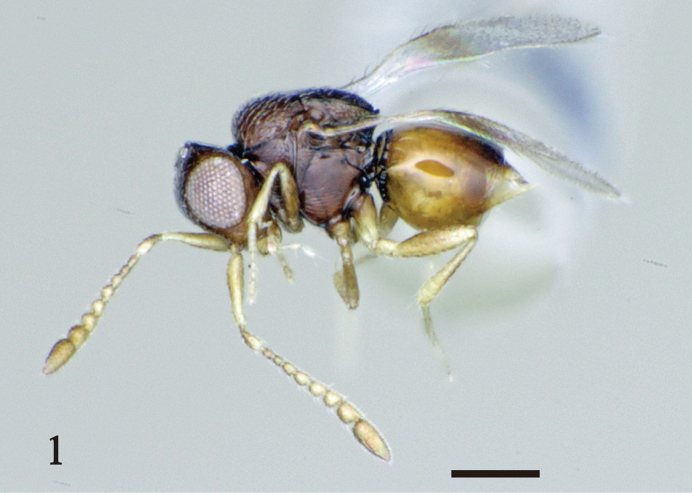
A female of *Aphanogmus flavigastris*. Scale bar: 100 μm.

**Figures 2–5.**
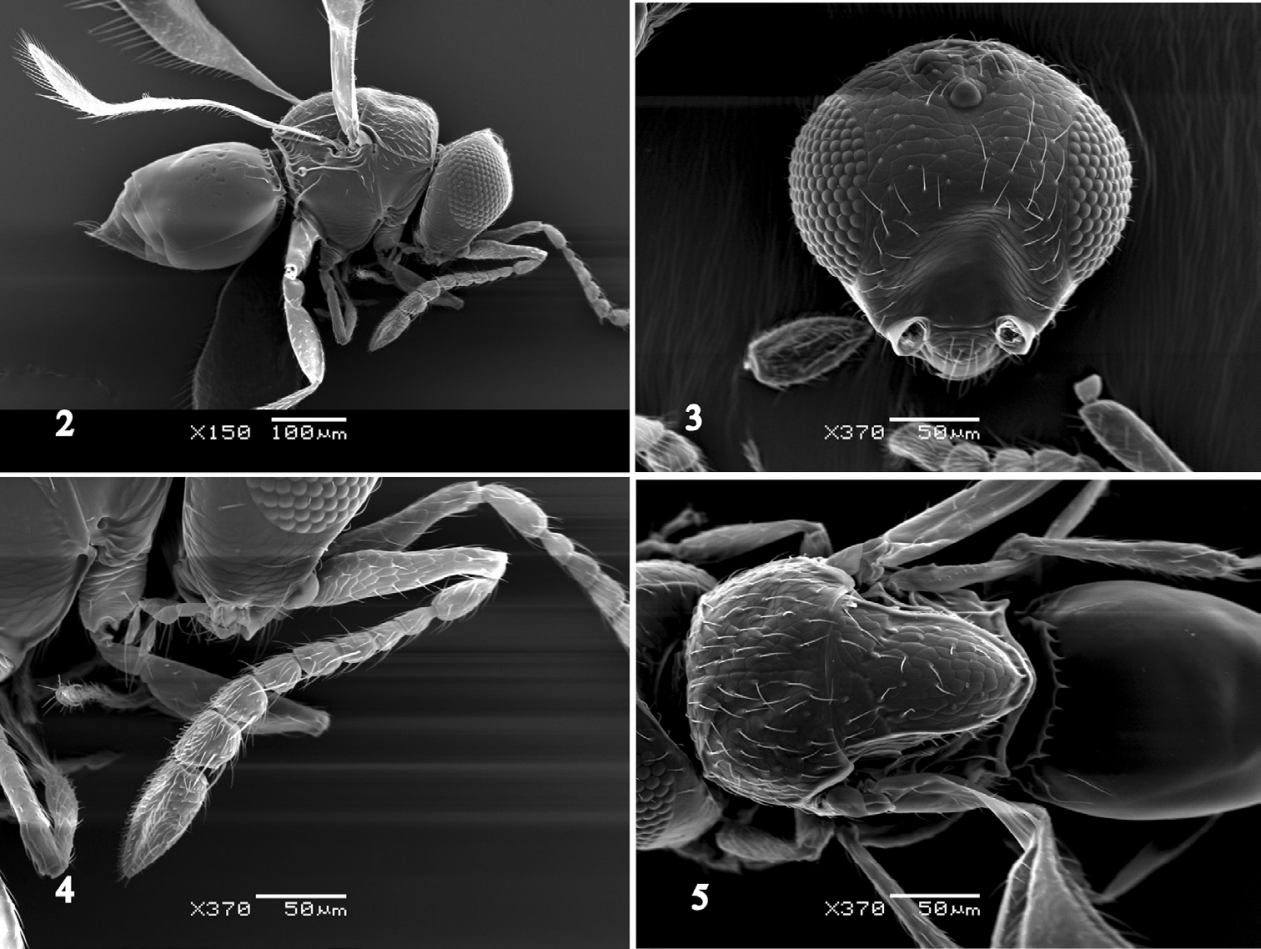
*Aphanogmus flavigastris*. **2** female body, lateral view **3** female head, frontal view; **4** female antenna, lateral view **5** female mesosoma and metasoma, dorsal view. Scale bars: **2**: 100 μm; **3–5**: 50 μm.

FEMALE. Body length 0.5–0.6 mm (Figs 1, 2). Head dark brown. Scape yellow; pedicel and all flagellomeres yellowish brown. Mesosoma dark brown. Fore wing with an infuscate area. Fore and mid coxae dark brown, sometimes yellowish in apical half; fore and mid femora yellow, sometimes brownish; hind leg and all tibiae yellow. Metasoma yellow, darker dorsally.

Head in dorsal view 1.5–1.7 times as wide as long, 1.2–1.4 times as wide as mesosoma; POL: OOL: LOL = 1.8: 1.5: 1.0. Head in frontal view (Fig. 3) 1.0–1.1 times as wide as high; malar space 0.3–0.5 times as long as eye height; lateral margin of torulus distinctly raised; intertorular carina distinct; frontal depression transversely reticulate; ocellar foveae absent; preocellar pit absent; facial pit absent; preoccipital furrow present and extends from anterior ocellus to occipital foramen; preoccipital carina absent; preoccipital lunula absent; occipital carina present; occipital depression absent; occiput smooth. Antenna (Fig. 4) 10 segmented; scape about 0.6 times as long as height of head, as long as distance between inner orbits; pedicel 2.0–2.5 times as long as flagellomere 1; the following segments gradually widened; flagellomere 7 about 2.0 times as wide as flagellomere 1; club 1 segmented.

**Figures 6–9.**
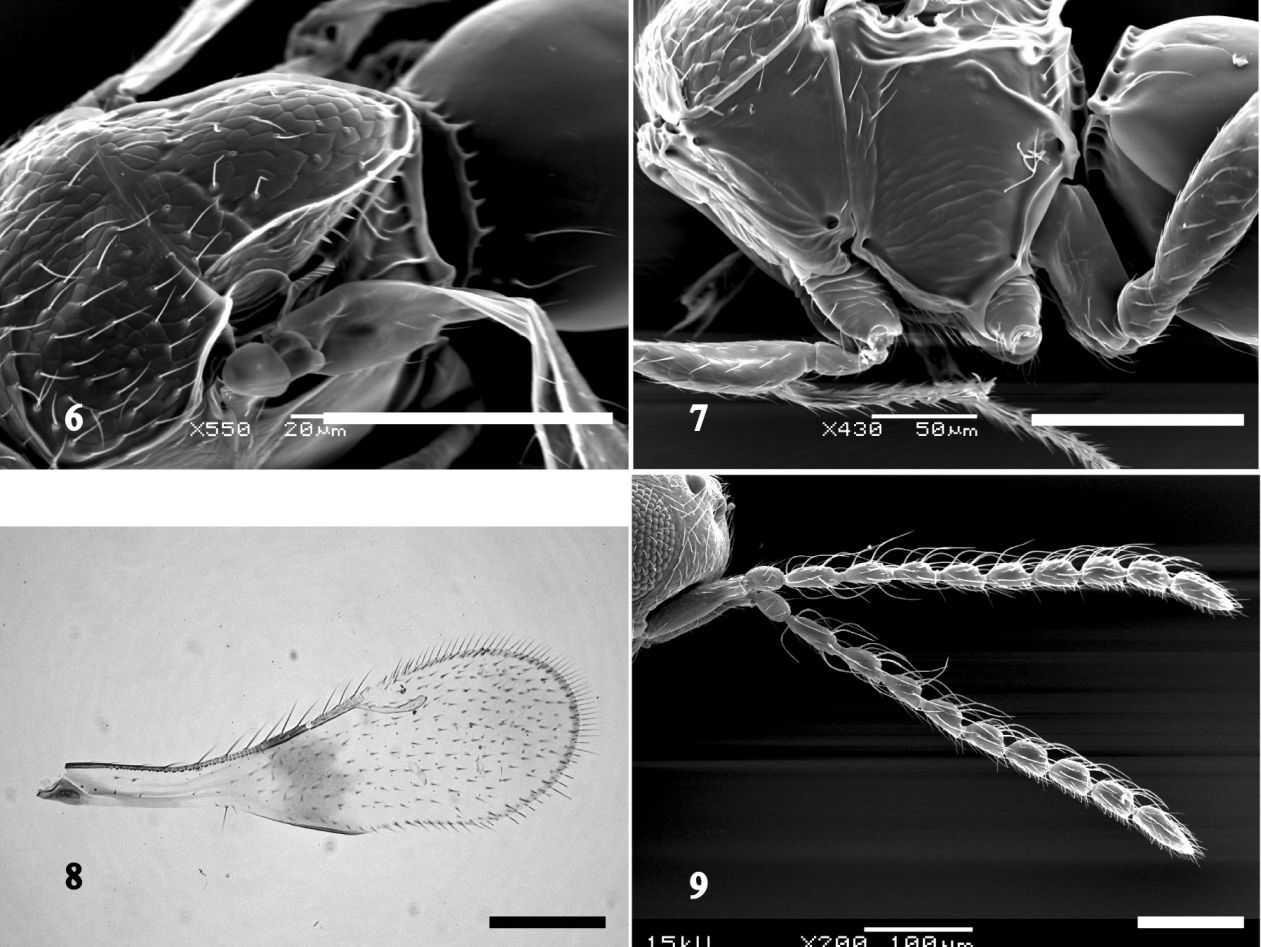
*Aphanogmus flavigastris*. **6** female scutellum, antero-dorsal view **7** female mesosoma, lateral view **8** female fore wing, upper surface **9** male antenna, lateral view. Scale bars: **6**: 20 μm; **7**: 50 μm; **8, 9**: 100 μm.

Mesosoma 1.2–1.4 times as long as wide; 1.3–1.5 times as high as wide; ventral pronotal pit distinct; mesoscutum reticulate, sparsely setose (Fig. 5); setal base slightly pustulate; median mesoscutal sulcus complete; notaulus absent; parapsidal line absent; interaxillar sulcus present; scutoscutellar sulcus angled medially, foveolate, continuous with interaxillar sulcus; dorsal axillar area and mesoscutellum sculptured as mesoscutum, with distinct lateral carina which connects posterior mesoscutellar sulcus (Fig. 6); mesoscutellum 1.4–1.6 times as long as wide; anterior mesopleural sulcus distinct (Fig. 7); anterior mesopleural area finely reticulate with several setae; dorsal mesometapleural carina straight; anterior mesopleural sulcus perpendicularly intersecting dorsal mesometapleural carina; metapleural carina distinct, extends near dorsal mesometapleural carina.

Fore wing about 3.0 times as long as wide, with a darkly pigmented band (Fig. 8); radial vein 1.4–1.5 times as long as marginal vein. Metacoxa bare dorsally; longitudinal metacoxal carina present at base.

Syntergum with distinct transverse carina anteriorly, smooth, with 2–3 setae anterolaterally, occupying more than half of total length of metasoma; longitudinal striae of syntergum absent.

MALE. Differs from female as follows: Antenna (Fig. 9) 11 segmented; flagellar setae long, about 2.0 times width of flagellomeres.

#### Distribution.

Japan.

#### Host insects.

*Feltiella acarisuga* and *Feltiella acarivora*. Usually one, occasionally two or three adults emerged from a single host cocoon.

##### Diagnosis

Evans et al. (2005) proposed the following three species groups based on characteristics of the mesosoma and metasoma:

*clavicornis* group: mesoscutal median furrow and metasomal basal carina absent. *tenuicornis* group: mesoscutal median furrow absent, metasomal basal carina present. *fumipennis* group: mesoscutal median furrow and metasomal basal carina present.

According to the morphological features of these species groups, the new species belongs to the *fumipennis* group, while *Aphanogmus fulmeki* and *Aphanogmus floridanus* that have been known as parasitoids of *Feltiella* species belong to the *clavicornis* group and *tenuicornis* group, respectively. Therefore, the new species can be distinguished from *Aphanogmus fulmeki* and *Aphanogmus floridanus*.

Among members of the *fumipennis* group, the new species shares the following characteristics with species in the *Aphanogmus hakonensis* complex *sensu* Polaszak and Dessart (1996): median mesoscutal sulcus present; dorsal axillar area and mesoscutellum with distinct lateral carina; syntergum with distinct transverse carina anteriorly. However, *Aphanogmus flavigastris* does not belong to the *Aphanogmus hakonensis* complex based on the following characters: fore wing with a darkly pigmented band (hyaline in *Aphanogmus hakonensis* complex); antenna of female with flagellomere 2–7 not transverse (transverse in *Aphanogmus hakonensis* complex).

The new species is most similar to *Aphanogmus inamicus* as it shares the following characters: median mesoscutal sulcus present; dorsal axillar area and mesoscutellum with distinct lateral carina; syntergum with distinct transverse carina anteriorly; fore wing with a darkly pigmented band; antenna of female with flagellomere 2–7 not transverse. However, *Aphanogmus flavigastris* can be distinguished from *Aphanogmus inamicus* by the following characters: club of antenna 1 segmented (3 segmented in *Aphanogmus inamicus*); lateral carina on dorsal axillar area and mesoscutellum more raised than that of *Aphanogmus inamicus*; longitudinal striae of syntergum absent (present in *Aphanogmus inamicus*); mesosoma dark brown (reddish yellow in *Aphanogmus inamicus*); infuscate area on fore wing smaller (from marginal vein to posterior margin of fore wing in *Aphanogmus inamicus*).

According to a key to the Palaearctic species of *Aphanogmus* (Szelényi 1940), the new species runs to *Aphanogmus fasciolatus* Förster based on the following characters: antenna clavate; club 1 segmented and longer than the preceding two segments combined; radial vein longer than marginal vein. However, the new species could be distinguished from *Aphanogmus fasciolatus* by having longer pedicel that is distinctly longer than flagellomere 1 while *Aphanogmus fasciolatus* has the pedicel that is shorter than flagellomere 1.

We need to monitor the seasonal abundance of *Aphanogmus flavigastris* for the successful application of *Feltiella* species, because its congener *Aphanogmus floridanus* that attacks *Feltiella acarivora* has been regarded to act as a negative force in controlling *Tetranychus urticae* Koch (Acari: Tetranychidae) on strawberry in California (Oatman 1985). Shimoda et al. (2016) recently developed a remarkable system for trapping *Feltiella* species and other predators of spider mites using pots of Brassica rapa Linnaeus var. perviridis L.H.Bailey (Brassicaceae), ‘komatsuna’ in Japanese, which bore *Tetranychus urticae*. They could rear an unidentified species of *Aphanogmus* from *Feltiella acarisuga* with the trapping system. This method may be useful to collect plenty of individuals of *Feltiella* and its parasitoids from ‘komatsuna’ in the fields. Further field surveys are needed to verify the efficacy of this method as a monitoring tool for *Aphanogmus flavigastris*.

## Supplementary Material

XML Treatment for Aphanogmus flavigastris
